# Development and evaluation of a test program for Y-site compatibility testing of total parenteral nutrition and intravenous drugs

**DOI:** 10.1186/s12937-016-0149-x

**Published:** 2016-03-22

**Authors:** Vigdis Staven, Siri Wang, Ingrid Grønlie, Ingunn Tho

**Affiliations:** 1 Hospital Pharmacy of North Norway Trust, Tromsø, Norway; 2 Department of Pharmacy, Faculty of Health Sciences, UiT The Arctic University of Norway, Tromsø, Norway; 3 Department for Medicinal Product Assessment, Norwegian Medicines Agency, Oslo, Norway; 4 Hospital Pharmacy, Haukeland University Hospital, Bergen, Norway; 5 Norwegian Medicines for Children Network, Bergen, Norway; 6 Department of Pediatrics, Haukeland University Hospital, Bergen, Norway; 7 School of Pharmacy, Faculty of Mathematics and Natural Sciences, University of Oslo, Oslo, Norway

**Keywords:** Parallel infusion, Incompatibility, Precipitation, Emulsion stability, Oil droplet size, Particle size, Particle content, Light obscuration, Zeta potential, Turbidity

## Abstract

**Background:**

There is no standardized procedure or consensus to which tests should be performed to judge compatibility/incompatibility of intravenous drugs. The purpose of this study was to establish and evaluate a test program of methods suitable for detection of *physical* incompatibility in Y-site administration of total parenteral nutrition (TPN) and drugs.

**Methods:**

Eight frequently used methods (dynamic light scattering, laser diffraction, light obscuration, turbidimetry, zeta potential, light microscopy, pH-measurements and visual examination using Tyndall beams), were scrutinized to elucidate strengths and weaknesses for compatibility testing. The responses of the methods were tested with samples containing precipitation of calcium phosphate and with heat destabilized TPN emulsions. A selection of drugs (acyclovir, ampicillin, ondansetron and paracetamol) was mixed with 3-in-1 TPN admixtures (Olimel® N5E, Kabiven® and SmofKabiven®) to assess compatibility (i.e. potential precipitates and emulsion stability). The obtained compatibility data was interpreted according to theory and compared to existing compatibility literature to further check the validity of the methods.

**Results:**

Light obscuration together with turbidimetry, visual inspection and pH-measurements were able to capture signs of precipitations. For the analysis of emulsion stability, light obscuration and estimation of percent droplets above 5 μm (PFAT5) seemed to be the most sensitive method; however laser diffraction and monitoring changes in pH might be a useful support. Samples should always be compared to unmixed controls to reveal changes induced by the mixing. General acceptance criteria are difficult to define, although some limits are suggested based on current experience. The experimental compatibility data was supported by scattered reports in literature, further confirming the suitability of the test program. However, conflicting data are common, which complicates the comparison to existing literature.

**Conclusions:**

Testing of these complex blends should be based on a combination of several methods and accompanied by theoretical considerations.

## Background

Critically ill hospitalized patients are often in need of many intravenous drugs, and a frequent problem is the lack of sufficient number of access sites or available lumen in multi-lumen catheters, to administer each product separately [1]. If a patient receives a continuous infusion of total parenteral nutrition (TPN), the infusion should be stopped and the line flushed before administration of other drugs in the same line [2, 3]. However, frequent stops may lead to under-nutrition, and the repeated flushing may be problematic with regard to the patient’s fluid balance [4]. In this situation it might be beneficial to co-administer drugs and TPN through a Y-site connector. Unfortunately, there is still a lack of documented experimental compatibility data [5, 6], and extrapolation of results can be difficult and risky [2, 3].

TPN admixtures as such are complex with a lot of possible physicochemical interactions [7–10]. Mixing TPN with drugs further complicates the picture [7, 10–14], potentially leading to chemical and/or physical incompatibility. Chemical degradation of ingredients (chemical incompatibility) [5, 15] is less relevant for Y-site administration because of the short contact time [5, 12, 14]. The focus of this paper is therefore on *physical* incompatibility that is precipitation of particles or growth of droplets leading to destabilization of the lipid emulsion [3, 11–14]. Both precipitates and large oil droplets can potentially be dangerous upon infusion. Cases of pulmonary emboli with fatale outcome have been reported after the administration of TPN containing calcium phosphate precipitates [16]. Also, deaths of neonatal patients caused by an incompatibility between ceftriaxone and calcium-containing products (ceftriaxone-calcium precipitate), have occurred [17]. The effects from injecting large (>5 μm) oil droplets are less clear, but animal studies have indicated that enlarged oil droplets can harm the lungs and liver [18, 19]. Furthermore, critically ill neonates receiving fat emulsions containing a high proportion of “large diameter tail” oil droplets, showed higher frequency of hypertriglyceridemia and poorer plasma clearance of lipids, compared to those receiving products with fewer large droplets [20]. There are also reports of emboli-like effects as a complication of intravenous fat emulsion administration [21, 22] although it is believed that the body can handle soft and flexible particles like oil droplets better than harder particles [23].

Since incompatibility might have serious consequences, compatibility data has to be based on solid documentation. However, there seems to be no consensus in literature to which tests should be performed to check incompatibility or what are the assessment criteria. Different studies apply different methods and the results are interpreted relatively to their test set. Some studies are based on only visual observations [12, 24], others include a few instrumental methods in addition [25–27], whereas some use more extensive set-ups with a combination of several methods [13, 28]. Assessment of possible precipitates are typically performed by one or more of the following approaches: visual examination with Tyndall light [11, 12, 24, 29] or other visual examination methods [13, 27, 30, 31], turbidimetric measurements [26, 29], colorimetric measurement [13], light obscuration (LO) [13, 31], dynamic light scattering (DLS) [27], microscopy [32] and pH-measurements [30–32]. Various methods have also been used to investigate parenteral emulsion stability: visual observation with [12] or without a centrifugation step [13, 25, 28, 32–35], determination of zeta potential [25, 28, 34, 36–38], measurement of dynamic surface tension [37], measurement of peroxide levels [28], pH-measurements [13, 25, 28, 33–35] and different droplet size measurement techniques. The latter techniques are e.g. microscopy [13, 28, 36, 37], DLS [13, 28, 33, 35–38], laser diffraction (LD) [25, 35, 36], flow cytometry [32], coulter counter [34, 36] and LO [13, 28, 33]. The list is not complete, but illustrates the diversity in methodology.

Also the acceptance criteria vary or may not be defined clearly. E.g. for electronically counting of particles, aiming at elucidating precipitates, some use the limits in the Pharmacopoeia for large volume parenterals; not more than 25 particles/ml ≥ 10 μm and 3 particles/ml ≥ 25 μm [13, 39]. Others also include smaller particles [31]. For turbidimetric measurements a change in turbidity by 0.5 NTU was defined as incompatibility by Trissel and Bready [29], and applied as specification in some compatibility studies [11, 26]. Also when it comes to the lipid emulsion stability, different criteria have been suggested, e.g. the many approaches to interpret stability based on microscopy data [13, 28, 36, 37, 40]. After inclusion of the droplet size requirements in the USP [41] (formally adopted in 2007 [42]), there are some official guidelines, even though equivalent requirements have not been adapted by the European Pharmacopeia. The USP standards states that the mean droplet diameter (MDD) in lipid injectable emulsions should be < 500 nm, measured by DLS or LD, and that the volume-weighted (V.W.) percentage of fat with droplet diameter above 5 μm (PFAT5) measured by LO, should be ≤ 0.05 % [41]. Although the USP limits are intended for “pure” lipid emulsions (10-30 % w/v emulsion) [41, 43], these limits have also been applied when the lipid emulsion is part of complex mixtures such as TPN [28, 44–46]. Extemporaneously prepared TPN admixtures may be less stable, and the limit of 0.05 % might be too strict; hence the use of PFAT5 < 0.40 % (one log higher) has been proposed as the acceptance criteria [47], although studies indicate that it is possible to formulate extempore TPN preparations that also fulfills the PFAT5 limit of the USP [45–47]. Driscoll and co-workers introduced PFAT5 > 0.40 % as a critical value characterizing unstable parenteral emulsions, and claimed that above this critical limit phase separation is likely to occur [33]. A PFAT5 limit of < 0.40 % has been applied as acceptance criteria in a compatibility study [13].

Attention has been drawn to the discrepancies between compatibility studies, and more standardization in conducting such studies and reporting from these are requested [5]. This work aimed to develop and evaluate a test program suitable to detect physical incompatibility of drugs and TPN in simulated Y-site administration. Methods frequently applied in the literature were scrutinized, and a selection of methods was tested in order to elucidate their strengths and weaknesses for compatibility testing purpose. The methods should be able to detect both signs of precipitation and emulsion destabilization. Incompatibility was defined as indications of an increase in size and/or number of particles, and/or an increase in lipid droplet size, both as compared to original, not-mixed samples of drug and TPN. To map the responses in the methods, TPN admixtures were subjected to stressful conditions (pH and temperature) to force physical incompatibility, i.e. precipitation and emulsion destabilization. To further evaluate the test program, three 3-in-1 TPN admixtures were tested with a selection of drugs. These drugs had previously been reported as compatible and some incompatible with TPN in the literature, or the existing data was conflicting. Based on the findings in the current study, a final selection of methods was chosen to constitute a test program. Suggestions for acceptance criteria were also formulated.

## Materials and methods

### Materials

Overview of the investigated 3-in-1 TPN admixtures, additives, drugs, dilution media and concentrations are listed in Tables 1 and 2. Four different paracetamol products were tested, and their composition is presented in Table 3. Formazin 4000 NTU, used to prepare formazin standards, was from Orion Application Solution (Thermo Scientific, Waltham, USA). Sodium hydroxide was from Sigma Aldrich (Seelze, Germany) and water was of Milli-Q quality (Millipore, Molsheim, France). Polystyrene standards (EZY-CAL^TM^/DUKE STANDARDS^TM^ Microsphere Size Standards, NIST Traceable Mean Diameter) were from Thermo Scientific (Fremont, USA).

**Table 1:** Overview of investigated 3-in-1 TPN admixtures and additives

Product type	Brand name	Manufacturer	Lot No.
3-in-1 TPN admixture	Olimel® N5E	Baxter	11B10N10
			11C27N10
			10J11N40
			12J03N12
			13C21N10
3-in-1 TPN admixture	Kabiven®	Fresenius Kabi	10BG8759
			10GD1825
			10GH5541
3-in-1 TPN admixture	SmofKabiven®	Fresenius Kabi	10GH6092
			10GM1499
			10HA2174
Trace elements	Tracel®	Fresenius Kabi	12ECB12
			12EFR02
			12FCB12
			12GHB16
			12GLB03
Vitamins water soluble	Soluvit®	Fresenius Kabi	10EE4869
			10EF6004
			10GD1632
			10GI7229
			10GM1588
Vitamins lipid-soluble	Vitalipid® Adult	Fresenius Kabi	10 EB2248
			10EL1064
			10GD1922
			10GK7978
			10GK7972
			10GF4227

**Table 2:** Overview of investigated drugs and concentrations

Drug	Manufacturer	Lot No.	Dilution media	Concentration after dilution (mg/ml)
Acyclovir sodium	Hospira	X171213AA X101213AB A101193AA A101213AA Y131213AB	Glucose 50 mg/ml	5 mg/ml
Ampicillin sodium	Bristol-Myers Squibb	2A00936 3E02641 0059774 3J01732 3L01792 3F02259 1E00687 1J00117 1C00905 0G56375 1A00661 0L60962 0J63206 3C02634	NaCl 9 mg/ml	50 mg/ml
	APP Pharmaceuticals	1K10AK		
	SAGENT Pharmaceuticals	P3667		
Ondansetron hydrochloride	Copyfarm	9278 18DI32602 18D350301	Glucose 50 mg/ml	0.2 mg/ml
	Fresenius Kabi	18F321601 18G207503 18G157402 18F321601 18E272402 18E183801 19E18380		
	Accord Healthcare	N08669 M13242		
Paracetamol	Bristol-Myers Squibb	0M44297 1E63971	Undiluted	10 mg/ml
	Fresenius Kabi	14GC20 14GF36 16FD0093 16EM0155		
	B. Braun	13486404 13487404 14382407		
	Actavis	14EE30 14EH47 16EM0155

**Table 3:** Composition of paracetamol formulations tested (Source: SmPC of the respective paracetamol products)

Pharmaceutical excipient	Bristol-Myers Squibb^a^/Actavis^a^	Fresenius Kabi^b^	B. Braun^c^
Cysteine hydrochloride monohydrate	+	+	
Disodium phosphate dihydrate	+		
Hydrochloric acid	+		
Mannitol	+	+	+
Sodium hydroxide	+		
Water for injections	+	+	+
Hydroxyethyl starch			+
Sodium acetate trihydrate			+
Sodium citrate dihydrate			+
Acetic acid, glacial			+
pH	5.5	5.0-7.0	4.5-5.5

a: tested with Olimel® N5E except for PFAT and laser diffraction measurements

b: tested with Kabiven® except for PFAT and laser diffraction measurements

c: tested with SmofKabiven®, all methods + with Olimel® N5E and Kabiven® with regard to PFAT and laser diffraction measurements

### Experimental design

Three commercially available 3-in-1 TPN products for central administration were studied. Drugs were chosen to demonstrate different types and degrees of incompatibilities with TPN. Pediatric patients is a vulnerable group, therefore concentrations of the drugs were selected to be clinically relevant for children from age of 2. The drug concentrations were selected in dialog with clinicians. Glucose 50 mg/ml was preferred as dilution media as this is recommended in children; sodium chloride 9 mg/ml was used when drugs were unstable in and/or incompatible with glucose according to the respective SmPC (Table 2). Tracel® and Vitalipid® Adult were chosen as additions of trace elements and fat soluble vitamins, instead of Peditrace® and Vitalipid® Infant. The latter products are used in the smallest children, but since Tracel® contains more types of trace elements and in higher concentration, adding Tracel® would represent the worst-case scenario. Vitalipid® Adult contains more of vitamin A and E, but less of K and D. Both vitamin products could have been used, but Vitalipid® Adult were chosen to “match” Tracel®, since both are used in the older children.

Aliquots of TPN were mixed with drug in sterile 50 ml polypropylene centrifuge tubes (VWR, Radnor, USA/Corning Incorporated, New York, USA) in three parallels of the mixing ratio 1 + 1. The order of mixing was TPN added to drug. The mixing was performed in a laminar airflow safety cabinet. Under the assumption that most incompatibilities worsen with increasing time, the samples were analyzed at two time points; immediately after mixing, which due to practical handling was within one hour and again four hours after mixing. However, the chance that e.g. a momentary precipitation would re-dissolve before detection cannot be excluded. Such a precipitate was regarded as less dangerous if infused, since it is likely to dissolve fast upon dilution in the circulation. Appropriate controls (pure TPN and pure drug solution) were used for comparison. TPN samples were also subjected to emulsion destabilization by heat (45 °C for 2 and 4 days) and addition of 0.1 M NaOH to induce alkaline pH, forcing precipitation of calcium phosphate. The analysis was performed under ambient laboratory conditions. The prepared TPN bags were stored in the fridge (4–8 °C) between sampling, and used before end of the maximal recommended storage time stated by the manufacturers (generally up to six days in fridge).

The milk-white appearance of TPN prohibits the direct assessment of potential precipitation. Therefore, sample preparation and the subsequent analysis were divided into two parts: assessment of potential precipitate (fat free TPN) and analysis of emulsion stability (all constituents of TPN present). The details of the methods are described below.

### Assessment of potential precipitate

#### Sample preparation

Two sample preparation approaches were evaluated: I) All three compartments of the 3-in-1 TPN-bag were blended, trace elements and vitamins added, and then mixed with drug. The mixtures were subjected to centrifugation (15 000 × g, 20 min, 23 °C) and the lipid layer on top was gently removed using a glass-pipette connected to a vacuum line [12]. II) The amino acid compartment and the glucose compartment were mixed, and Milli-Q-water was added to replace the lipid emulsion [48]. Trace elements were added, but vitamins were omitted to avoid disturbances from strong color, especially with regard to the visual examination [15]. This TPN-derivative is further referred to as TPN_aq_ to emphasize the lipid-free alternative [48]. Both TPN_aq_ and drugs were filtered 0.22 μm before mixing.

#### Visual examination using Tyndall light

Visual examination was carried out to identify Tyndall effect and light scattering from potential precipitated particles. Samples were mixed in sterile, Milli-Q-water rinsed, 100 × 24 × 0.9-1.0 mm flat-bottom glass tubes (Scherf Präzision Europa GmbH, Meiningen, Germany) instead of the centrifuge tubes. Three different light sources were applied: I) a 75 watt halogen light bulb in a desk lamp, covered with an aluminum plate with a 1.5 cm diameter hole to focus the light, II) a red pocket laser pointer (630–650 nm, max output <1 mW), and III) a fiber optic light source (Schott KL 1600 LED, Mainz, Germany). The samples were studied against a black background in a dark room as described earlier [48].

### Light obscuration analysis

Sub-visual particles were counted using LO (Accusizer 780 Optical Particle Sizer, Nicomp PSS, Santa Barbara, USA). The performance of the instrument was verified with polystyrene microsphere standards. The principle of LO is the momentary blockage (large particles) or scattering (small particles) of light when particles pass through a sensing zone illuminated by a laser beam. This creates a pulse that translates to a specific particle size when compared to an established calibration curve [49]. Given that the concentration is sufficiently dilute, this is an optical single particle counting technique where the number and size of individual particles are estimated one at a time [49]. Too high concentrations can lead to multiple particles (clusters) being sized and counted as one particle. The sensor was LE-400-05 in summation mode, measuring particles from 0.5 to 400 μm. The background count of the centrifugation tubes was below 100 particles/ml ≥ 0.5 μm.

15 ml of sample was measured undiluted to avoid dissolution of precipitate. The total particle count/ml ≥ 0.5 μm and the amount of particles ≥ 5, 10 and 25 μm per ml [39] were determined. In addition the particle content of TPN_aq_ with increasing amount of 0.1 M NaOH was measured. Also, a sample of known incompatibility (acyclovir: TPN_aq_ 1 + 1) was measured every 30 min after mixing up to 4 h.

### Turbidity measurements

Two methods were employed to evaluate turbidity, a ratio turbidimeter (2100Qis Turbidimeter, Hach Lange GmbH, Düsseldorf, Germany) measuring in formazin nephelometry units (FNU) and an UV–VIS spectrophotometer (Agilant 8453 UV-visible Spectroscopy system, Agilent Technologies, Santa Clara, USA) measuring the % transmittance in 1 cm quartz cuvettes at 550 nm [50]. Relative transmittance was calculated from T/T_0_, where T is the % transmittance in samples and T_0_ is the % transmittance in pure TPN_aq_ or Milli-Q-water respectively [50]. Ratio turbidimetry measures the scattering of incident light at different angles, formed due to light interacting with particles present in the samples [51]. In an UV–VIS spectrophotometer the incident light is attenuated by particles present, and the remaining light reaching the detector at 180° relative to the incident light path is measured [51]. Both instruments were validated with formazin standards of 3, 6, 18 and 30 NTU as described in Ph. Eur. [52].

The turbidity (FNU) of TPN_aq_ with increasing alkalinity (i.e. calcium phosphate precipitation) and TPN_aq_ mixed with acyclovir (same as for LO: every 30 min after mixing up to 4 h), were measured. To further check the correlation between the methods, low turbidity samples (<1 FNU) were measured with both instruments (ampicillin alone, ampicillin with TPN_aq_, paracetamol alone, paracetamol with TPN_aq,_ and ondansetron with TPN_aq_).

### pH measurements and theoretical consideration

The pH of samples was measured with a pH meter (Metrohm 744 pH Meter, Metrohm AG, Herisau, Switzerland) calibrated with buffers of pH 4.00 and 7.00. For samples involving alkali drugs calibration buffers of pH 10.00 was included in the calibration. A theoretical evaluation of solubility and compatibility was also performed.

### Emulsion stability analysis

#### Sample preparation

All three chambers of the TPN-bags were mixed and vitamins and trace elements were added. Drugs, but not TPN, were filtered 0.22 μm. In addition to drug: TPN-samples, heat treated TPN as described above, were analyzed.

#### Dynamic light scattering

The intensity weighted (I.W.) MDD and polydispersity index (PI) were estimated using DLS (Submicron Particle Sizer Modell 370, Nicomp PSS, Santa Barbara, USA). The principle of DLS is the measurement of temporal fluctuations in scattered light caused by the Brownian motion of small particles in dispersion. From this the hydrodynamic diameter of the particle distribution can be deduced. DLS measures particles from nanometers to a few microns [49]. Prior to measurements the performance of the instrument was verified with polystyrene microspheres. The samples were diluted in Milli-Q-Water in disposable borosilicate glass culture tubes of 6x50 mm (Kimble Chase, Rockwood, USA) to an intensity of 250–350 KHz and measured.

### Laser diffraction

The V.W. MDD and V.W. percent of particles below 500 nm and 1 μm, and above 5 and 10 μm, were estimated using LD (Mastersizer 2000 and Hydro 2000G sample dispersion unit) (Malvern Instruments, Worcestershire, UK). LD measures the angular light scattering pattern of particles in dispersion. Light is scattered differently relative to particle size; small angles for large particles and large angles for small particles. Based on the scattered pattern the particle size distribution can be estimated, and typically reported on a volume basis. The measuring range is broad, from nanometers to millimeters [49]. Like DLS, LD is an ensemble technique, i.e. the collective scattering from all the particles contribute to the signal. This is in contrast to single optical particle counting like LO. The samples were added to the sample dispersion unit, filled with Milli-Q-water. The sonication was turned off to avoid breaking up large droplets. The following parameters were applied: absorbance: 0.001 and refractive index: 1.46. The instruments performance was verified with polystyrene microspheres.

### Light obscuration

To investigate changes in the large diameter tail of the fat emulsion, LO was used. The sensor was set in extinction mode and the detection threshold was 1.80 μm. Dilution of samples was performed in a 40 ml glass beaker with Milli-Q-water. A micropipette was used for sampling. Samples were diluted to concentrations below the instrument’s coincidence limit of 9000 particles/ml, using dilution factors of 1:400–8000 (sample:water). The samples were stirred for 60 seconds prior to measurements [53]. The sample withdrawal from the diluted emulsions was 15 ml. The counts were distributed over 128 channels, and the equivalent spherical volumes of the oil droplets were calculated. The density of oil used in calculations was 0.92 g/ml and the final fat composition varied between 0.038 to 0.040 g/ml (including fat from Vitalipid® Adult) depending on the respective TPN product (Table 4). The volume weighted percentage of fat (PFAT) greater than 2, 5 and 10 μm, were estimated to look for active growth in different size fractions of the large diameter tail. The calculations were done as described in the literature [33, 53].

**Table 4:** Composition of the three 3-in-1 TPN admixtures per liter

	Olimel® N5E TPN_aq_/TPN	Kabiven® 1900 kcal TPN_aq_/TPN	SmofKabiven® 1600 kcal TPN_aq_/TPN
Lipids total (*g*)	-/39.2	-/38.2	-/36.9
Olive oil (%)	-/80	-/-	-/25
Soybean oil (%)	-/20	-/100	-/30
MCT (%)	-/-	-/-	-/30
Fish oil (%)	-/-	-/-	-/15
Glucose anhydrous (*g*)	113.9/112.7	96.5/95.6	124.9/123.3
Amino acids total (*g*)	32.6/32.3	32.8/32.5	50.1/49.4
Alanine (*g*)	4.7	4.6	7.0/6.9
Arginine (*g*)	3.2	3.3/3.2	6.0/5.9
Aspartic acid (*g*)	0.9	1.0	-
Glutamic acid (*g*)	1.6	1.6	-
Glycine (*g*)	2.3/2.2	2.3/2.2	5.5/5.4
Histidine (*g*)	1.9	2.0	1.5
Isoleucine (*g*)	1.6	1.6	2.5
Leucine (*g*)	2.3/2.2	2.3/2.2	3.7
Lysine (*g*)	2.6/2.5	2.6	3.3
Methionine (*g*)	1.6	1.6	2.1
Phenylalanine (*g*)	2.3/2.2	2.3/2.2	2.5
Proline (*g*)	1.9	2.0	5.6/5.5
Serine (*g*)	1.3	1.3	3.3/3.2
Taurine (*g*)	-	-	0.5
Threonine (*g*)	1.6	1.6	2.2
Tryptophan (*g*)	0.5	0.5	1.0
Tyrosine (*g*)	0.1	0.1	0.2
Valine (*g*)	2.1	2.1	3.1/3.0
Sodium (*mmol*)	34.6/34.3	30.8/30.6	40.1/39.5
Potassium (*mmol*)	29.7/29.4	23.2/22.9	30.1/29.6
Magnesium (*mmol*)	4/3.9	3.9/3.8	5.0/4.9
Calcium^a^ (*mmol*)	3.5/3.4	1.9/1.9	2.5/2.5
Phosphate^b^ (*mmol*)	11.9/14.9	6.8/9.7	9.9/12.7
Acetate (*mmol*)	36.1/35.8	37.6/37.3	104.9/103.5
Chloride (*mmol*)	44.6/44.1	44.9/44.4	34.7/34.3
Zink (*mmol*)	-	-	0.04
Sulphate (*mmol*)	-	3.6/3.8	5.0/4.9
α tocoferol	-	-	n.s
Phospholipids from egg	n.s	n.s	n.s
Glycerol	n.s	n.s	n.s
Sodium oleate	n.s	-	n.s
Water for injections	n.s	n.s	n.s
pH	6.4	5.6	5.6
Tracel®^c^ (ml)	10/9.9	9.7/9.6	13.5/13.4
Soluvit®^c^ (vials)	-/1	-/1	-/1.3
Vitalipid® adult^c^ (ml)	-/9.9	-/9.6	-/13.4

a: calcium chloride as calcium source

b: from sodium glycerophosphate, the emulsion and Vitalipid® adult

c: additives

Excipients other than for pH adjustment are listed

*n.s*. the quantity is not stated

Source: SmPC from the respective TPN products

### Microscopy

The emulsion droplets were studied in a light microscope (LM) under 1000 × magnification (Zeiss Axioscope 451485 Light microscope, Carl Zeiss AG, Jena, Germany). A small sample-droplet was placed upon a slide, and covered with a slide with immersion oil. The preparation was studied and photographed (Cannon EOS 300D Digital, Canon, Tokyo, Japan). Due to the lack of a measuring ocular of adequate fineness, a 5 μm scale was added to the images afterwards with the aid of reference images of 5 μm and 10 μm polystyrene microsphere size standards.

### pH-measurements and theoretical consideration

The pH was measured, and theoretical considerations regarding emulsion stability were performed.

### Zeta potential measurements

The zeta potential of the samples was determined by laser doppler micro-electrophoresis (Zetasizer Nanoseries Nano Z) (Malvern instruments, Worcestershire, UK) using a folded capillary sample cell (DTS 1060, Malvern, Worcestershire, UK). In this technique, the emulsion is diluted in a medium that is exposed to an electric field, which causes movement of the charged emulsion droplets to the oppositely charged pole. At the same time the sample is illuminated with a laser beam. The movement causes a shift in the frequency of scattered light (“Doppler shift”), which is used to determine the velocity of the movement of the droplets, the electrophoretic mobility and the zeta potential [49]. The samples were diluted 1:1000 (sample:water) in distilled water [25]. The calibration of the instrument was checked with a zeta potential transfer standard (−68 mV ± 6.8 mV) (Malvern Instruments, Worcestershire, UK).

### Statistical analysis

For group comparison of the results, one-way ANOVA followed by Tukey’s *Post Hoc* test was applied, α = 0.05 (Minitab® 16 Statistical Software, Minitab Inc., USA). Student’s t-tests (SPSS) were also applied to compare two means.

## Results and discussions

### Selection of test materials

Since the purpose of this study was to establish a set of methods suitable for the detection of possible Y-site incompatibility of TPN and i.v. drugs, test materials were selected for which there were existing compatibility reports available in literature. Acyclovir is known to precipitate when mixed with TPN [11, 12, 24, 25]. This drug was included as a positive control on precipitation. Ampicillin has shown conflicting results in the literature, some has reported formation of precipitate [24, 30] whereas others have concluded that the drug is compatible with TPN [11, 12]. Ondansetron has been reported to destabilize the emulsion [12], although other studies have reported no change [13, 25, 54]. There are very few studies on paracetamol and TPN, we found only one available report and it concluded with compatibility [13]. It should be notated noted that paracetamol is available in different formulations in generic products, and it was also included as a realistic example of dealing with possible effects related to generic formulation diversity.

The TPN products all had different fat emulsions (Table 4). The amino acid content of Olimel® N5E and Kabiven® was similar, whereas the content of SmofKabiven® was higher and of different composition. Olimel® N5E had the highest content of calcium and phosphate, and Kabiven® the lowest. According to the manufacturers’ specifications, all three TPN products could tolerate more electrolytes, meaning that they were not stressed to their maximum. The pH was 0.8 pH units lower in Kabiven® and SmofKabiven® as compared to Olimel® N5E (Table 4). Altogether, the selected products represented some variability in TPN admixture composition.

### Assessment of potential precipitate

**Fig. 1:**
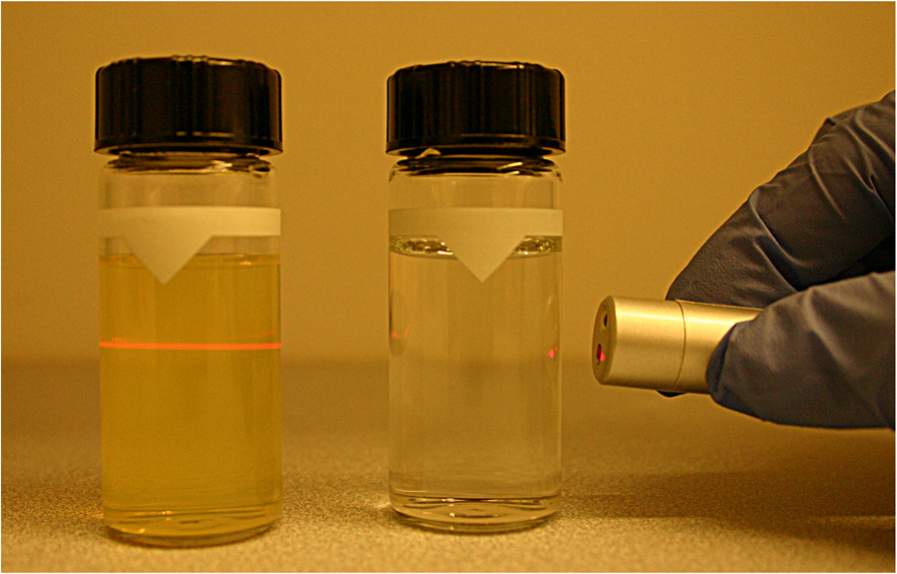
Illumination with a Tyndall beam of samples prepared by different strategies. Left: approach I) centrifugation of 3-in-1 TPN and removal of supernatant, Right: approach II) lipid compartment replaced with Milli-Q water (TPN_aq_). The centrifuged sample had a turbidity reading of 117 FNU and TPN_aq_ 0.08 FNU

Methods suitable to assess precipitates should be able to detect visual and sub-visual particles if present, and to estimate their particle size and number. In order to give the correct result, the appropriate sample preparation method is foremost important. Samples prepared by the centrifugation method showed that it was difficult to remove all traces of lipids and emulsifiers from the aqueous phase, resulting in samples with an inherent Tyndall effect (see Fig. 1). This was causing problems for further assessment of precipitation for all methods. An older report suggests diluting the remaining aqueous-phase (after removal of lipid layer on top resulting from the centrifugation) with water to ease the visual examination of particles (presumably by diluting the cloudiness caused by the remnants of the oil phase) [12]; however this might result in re-dissolving precipitated particles and should be avoided. Another concern may be that microprecipitate adhere to the fat droplets, and therefore will be removed together with the lipids and remain undetected with this set-up.

The sample preparation where the lipid compartment was replaced by Milli-Q water (TPN_aq_) gave a clear solution with very low Tyndall effect (Fig. 1). The disadvantage of this approach might be that since the lipid compartment and vitamins are omitted the potential impact of them on the precipitation will not be captured. In the case of poorly water-soluble drugs the lipid compartment might contribute to keeping the drug in solution [55], and removing the solubilizing aid would result in precipitation. This would give a false positive result, which in compatibility evaluation is less serious than the opposite. Adding lipid emulsion might increase the pH of TPN, since pure emulsion has pH values between 6.0 and 9.0 [43], therefore it is important to know the pH in both the TPN_aq_ and TPN. However, the buffer capacity of the amino acids will likely prevent a large difference [7, 9]. Mirtallo listed drugs showing different compatibility in 2-in-1 versus 3-in-1 TPN mixtures [55] but the discrepancies were mostly due to emulsion disruption and not precipitation [56]. When it comes to the vitamins, it has been reported that Vitamin C might degrade to oxalic acid and precipitate with calcium [9, 10]. Nevertheless, only one report of drug forming precipitate in Y-site with multivitamins (pantoprazole sodium) was identified in the Handbook on injectable drugs [56], although a scarcity of studies can contribute to the lack of reports. Finally, similar sample preparation approaches have been suggested earlier in other compatibility studies of TPN and drugs [13, 26]. The results from the assessment of precipitation with different methods are summarized in Table 5.

**Table 5:** Results from assessment of precipitation in TPN_aq_

TPN_aq_	Drug	Time after mixing *h*	*Light obscuration*				*Visible particles and*/*or Tyndall effect* (+/−)	*Turbidity* (*FNU*)	*pH*
			*Particles*	*Particles*	*Particles*	*Particles*			
			≥*0.5* μ*m*/*ml*	≥*5* μ*m*/*ml*	≥*10* μ*m*/*ml*	≥*25* μ*m*/*ml*			
None	Acyclovir alone	-	238 ± 58	3 ± 1	1 ± 0	0 ± 0	-	0.07 ± 0.00	10.03
	Ampicillin alone	-	144 ± 50	1 ± 1	0 ± 0	0 ± 0	^c^+	0.94 ± 0.06	8.87
	Ondansetron alone	-	n.d.	n.d.	n.d.	n.d.	-	0.08 ± 0.01	4.21
	Paracetamol alone^a^	-	n.d.	n.d.	n.d.	n.d.	^c^+	0.59 ± 0.01	5.33
	Paracetamol alone^b^	-	n.d.	n.d.	n.d.	n.d.	-	0.08 ± 0.00	5.42
Olimel® N5E	Fresh without drug	-	119 ± 63	2 ± 2	1 ± 1	0 ± 1	^d^+	0.10 ± 0.03	6.30
	Acyclovir	0	810 ± 190	37 ± 17	6 ± 3	0 ± 0	++	0.38 ± 0.37	7.80
		4	6763 ± 1148	4051 ± 1578	2513 ± 1470	691 ± 679	+++	138.09 ± 211.14	7.63
	Ampicillin	0	485 ± 412	4 ± 2	1 ± 0	0 ± 0	^e^+	0.43 ± 0.12	7.93
		4	9550 ± 2285	1 ± 1	0 ± 0	0 ± 0	^e^+	0.49 ± 0.18	7.81
	Ondansetron	0	246 ± 102	1 ± 1	0 ± 0	0 ± 0	-	0.08 ± 0.02	6.22
		4	287 ± 84	2 ± 2	0 ± 0	0 ± 0	-	0.08 ± 0.01	6.15
	Paracetamol	0	242 ± 104	9 ± 3	1 ± 2	0 ± 0	-	0.10 ± 0.00	6.19
		4	153 ± 75	11 ± 6	2 ± 1	0 ± 0	-	0.09 ± 0.01	6.17
Kabiven®	Fresh without drug	-	16 ± 2	0 ± 0	0 ± 0	0 ± 0	^d^+	0.13 ± 0.03	5.59
	Acyclovir	0	3271 ± 2421	410 ± 361	128 ± 98	22 ± 16	++	2.16 ± 1.80	7.18
		4	7520 ± 229	6432 ± 211	5163 ± 171	2540 ± 33	+++	683.50 ± 135.06	7.10
	Ampicillin	0	157 ± 101	2 ± 1	1 ± 1	0 ± 0	^e^+	0.61 ± 0.22	7.77
		4	3851 ± 2300	2 ± 1	1 ± 1	0 ± 0	^e^+	0.64 ± 0.18	7.67
	Ondansetron	0	103 ± 57	5 ± 1	2 ± 1	0 ± 0	-	0.11 ± 0.01	5.55
		4	88 ± 2	2 ± 2	1 ± 2	0 ± 0	-	0.12 ± 0.02	5.55
	Paracetamol	0	128 ± 125	1 ± 0	0 ± 0	0 ± 0	-	0.10 ± 0.02	5.56
		4	36 ± 23	0 ± 0	0 ± 0	0 ± 0	-	0.10 ± 0.01	5.56
SmofKabiven®	Fresh without drug	-	171 ± 100	5 ± 5	3 ± 3	1 ± 1	^d^+	0.11 ± 0.03	5.50
	Acyclovir	0	650 ± 278	23 ± 8	4 ± 1	0 ± 0	++	0.11 ± 0.02	6.48
		4	7088 ± 456	4043 ± 745	2177 ± 697	290 ± 139	+++	86.83 ± 36.60	6.47
	Ampicillin	0	505 ± 185	7 ± 6	2 ± 2	0 ± 0	^e^+	0.26 ± 0.04	7.64
		4	2531 ± 1196	3 ± 2	1 ± 1	0 ± 0	^e^+	0.27 ± 0.04	7.48
	Ondansetron	0	172 ± 73	1 ± 1	0 ± 0	0 ± 0	-	0.09 ± 0.01	5.47
		4	231 ± 110	1 ± 0	1 ± 0	0 ± 0	-	0.09 ± 0.01	5.49
	Paracetamol	0	325 ± 73	2 ± 0	1 ± 0	0 ± 0	^f^+	0.32 ± 0.01	5.38
		4	341 ± 182	1 ± 0	1 ± 0	0 ± 0	^f^+	0.33 ± 0.01	5.38

a: B. Braun; b: formulations other than B. Braun’s; c: Tyndall effect in pure drug solution, no particles; d: very weak Tyndall effect in pure TPN_aq_ solution, no particles; e: might be a combination of Tyndall effect in pure drug solution and very fine particles; f: probably due to Tyndall effect in pure drug solution and not precipitation

Mean ± standard deviation (*n* = ≥ 3)

### Visual examination using Tyndall light

Only samples containing acyclovir showed clear signs of precipitation visually. Immediately after mixing with TPN_aq_ small needle shaped particles could be seen using Tyndall light. After four hours the precipitation was obvious also for the unaided eye in normal light (Table 5). For ampicillin the precipitation was not clearly detected by visual examination because this drug also displayed a Tyndall effect alone. Also one of the paracetamol formulations showed this property (Table 3 and Table 5). The observations of Tyndall effect in the original drug solutions made it difficult to perceive possible additional particles or haze due to incompatibility. This has been discussed in more detail in a study on the validity and reliability of visual examination with Tyndall light for compatibility testing [48].

### Light obscuration

**Fig. 2:**
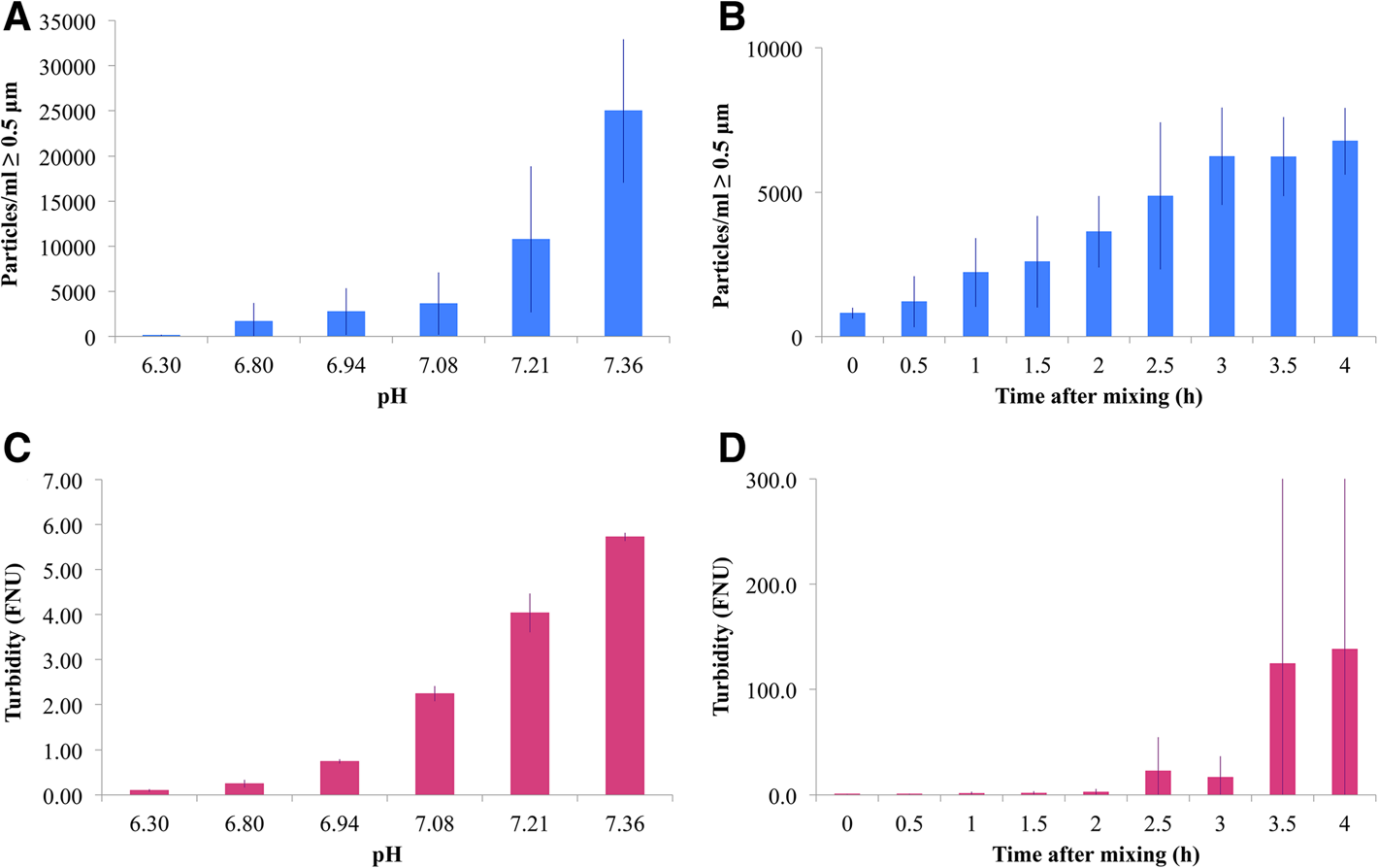
Results from light obscuration (**a**-**b**) and turbidimetric measurements (**c**-**d**) on two different precipitation reactions. **a** increasing number of particles larger than 0.5 μm per ml as the pH of TPN_aq_ increases from addition of 0.1 M NaOH (*n* > 3). The first column represents TPN_aq_ without added NaOH; **b** particles larger than 0.5 μm per ml in samples of TPN_aq_ and Acyclovir (1 + 1) at different time points after mixing (*n* = 3); **c** increasing turbidity with increasing pH of TPN_aq_ (*n* = 2). The first column represents TPN_aq_ without added NaOH; **d** turbidity in samples with TPN_aq_ and Acyclovir (1 + 1) at different time points after mixing (*n* = 3)

As expected, low total particle counts were detected in pure TPN_aq_, i.e. close to the background of the tubes (Table 5). An increasing, although varying, number of particles (≥0.5 μm/ml) were detected in samples of TPN_aq_ as they were titrated with 0.1 M NaOH to force precipitation (Fig. 2a). Once alkali was added the mean particle count increased markedly to over 1000 counts per ml. Increasing the pH above the pK_a2_ of phosphoric acid at 7.2 [57], a jump in the particle content could be observed. Following acyclovir + TPN_aq_ every 30 min, from immediately after mixing up to four hours, the particle counts increased continuously, although the variation was large. Immediately after mixing, the particle content was already increased to almost 1000 particles/ml.

The particle counts indicated a massive growth in number of particles for both acyclovir and ampicillin after mixing with all TPN_aq_ (Table 5). Immediately after mixing the detected particle numbers showed high variability, but there was a strong increase within the four hours of the study (Table 5 and Fig. 2b). As mentioned above, whether ampicillin is compatible or not with TPN is disputed in literature. However, in the current study a clear indication of precipitation taking place was found for all three TPN_aq_ solutions investigated: The highest amount of particles was found in Olimel® N5E, which might be an effect of pH (TPN with the highest pH before the addition of drug with alkali pH) or caused by the higher content of calcium and phosphate (Table 4). LO seemed to be a sensitive method for detecting ongoing precipitation.

Some factors can affect the results: Micro-bubbles may be counted as particles [58], particles adhering to container surfaces might not be counted [9, 11, 14], and some types of particles might be undercounted or others give too high counts, causing artifacts [59]. Therefore, some caution and experience in interpreting LO results are necessary. The smallest particles (<2 μm) are counted less accurately [58]. We do have experience with precipitation in other samples not part of this study, that was detected by visual examination and turbidimeter and not by LO, possibly because the particles were smaller than the detection limit of LO instrument. Due to their numerous amounts and collective light scattering, they could be seen in Tyndall light and by turbidity measurements. Furthermore, large particles present in low numbers might not be detected by LO [59], and other methods, such as visual examinations, might be more appropriate. Conversely: there are situations where LO counts many particles without these being detected with turbidimetri or Tyndall light. It clearly depends on the type of particles and other influencing factors. The main advantages of LO is that it is rapid and provides numerical and objective counts of particles as compared to visual methods [31, 48, 57].

### Turbidity measurements

Increased turbidity (FNU) was detected in samples of TPN_aq_ with forced precipitation (Fig. 2c), approaching 0.2-0.3 FNU after the initial adding of alkali. Approaching pH 7.2, the turbidity was around 4 FNU. The turbidity increased over time in mixtures of acyclovir and TPN_aq_ (Fig. 2d), however, the measured turbidity showed increasing variation due to varying degree of sedimentation of the larger and more heavy particles. Immediately the turbidity was 0.38 FNU increasing to 0.44 after 30 min.

**Fig. 3:**
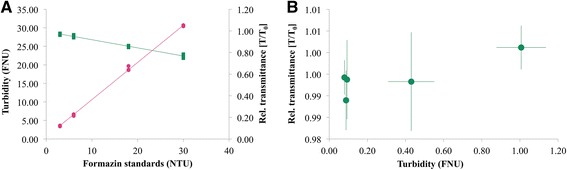
Turbidity measurements by two methods. **a** Turbidity of formazin standards (3, 6, 18 and 30 NTU) measured with turbidimeter (FNU in pink) and UV–vis spectrophotometer (relative transmittance in green) T_0_ = Milli-Q-water (*n* = 3). **b** Relationship between measured relative transmittance and FNU of samples of drug: TPN_aq_ (1 + 1) and pure drug solutions. T_0_ = TPN_aq_ (*n* = ≥3)

As shown in Fig. 3a both the spectrophotometer and the turbidimeter gave a linear response measuring the increasingly turbid formazin standards. Nevertheless, looking closer at samples of low turbidity (<1 FNU) the measured relative transmittance (T/T_0_) showed more unpredictable results, not conforming to FNU (Fig. 3b). Spectrophotometers are not recommended for samples with very low turbidity, but are more suitable at higher turbidity values (optimal range 20–1000 units) [51]. As it has been suggested to define incompatibility as a change in turbidity of 0.50 NTU [29], a turbidimeter should be preferred in order to detect such small differences. [FNU is equivalent to NTU up to 40 NTU [52]].

The mixed samples containing ampicillin showed higher turbidity values than the rest of the samples, indicating the presence of a precipitation (Table 5). However, ampicillin alone had an even higher value. Ampicillin is a powder before reconstitution; therefore it might be speculated that undissolved drug particles were causing this. However, the drugs were filtered 0.22 μm before mixing, reducing this, and LO measurement of pure drug solution did not show particle growth over time (data not shown). The Tyndall effect observed could be due to fluorescence of the drug [48, 60] or that ampicillin form micelles in aqueous solution [61]. When in doubt, comparing samples to controls of drug diluted in Milli-Q-water might be useful for drugs with inherent turbidity. Fox et al. compared samples to TPN:sterile water and drug:sterile water (ratio 1 + 1) controls in a compatibility study [26]. The turbidity of samples with paracetamol and ondansetron remained low after mixing with TPN_aq_ except for mixtures with TPN_aq_ from SmofKabiven®. The latter seems to be attributed to the paracetamol formulation (inherent Tyndall effect) rather than a result of mixing with SmofKabiven®. Since the tests were performed over an elevated period of time, drugs from different manufacturers were used depending on what was available from the local hospital pharmacy, but only paracetamol varied in excipient composition (Table 3). The formulation from B. Braun showed high turbidity values, which might be caused by hydroxyethyl starch forming a colloidal suspension. The turbidity method and the visual examination captured the difference in formulation between these drug products.

### pH measurement and theoretical consideration

Since most drugs are weak acids or bases, their solubility will be pH-dependent. The final pH in a mix of TPN and drug will depend on the pH of the formulations, buffering capacity and the concentrations. TPN products possess a large buffer capacity, because of the content of amino acids and acetate and can often withstand large changes in pH [7, 9].

In the current study, acyclovir and ampicillin stands out with quite alkaline pH values (Table 5), which increase the risk of calcium phosphate precipitation [57], or precipitation of the unionized drug upon mixing with the less alkaline TPN_aq_. Acyclovir is sparingly to slightly soluble in water, whereas the sodium salt is soluble 1 in 10 of water [62]. The pKa value of the proton donating group is 9.3 [63]. After mixing with TPN_aq_ the pH value is too low for the drug to be sufficiently ionized, and at the same time high enough to cause calcium phosphate precipitation. Hence, the observed precipitation can be a result of both.

Ampicillin is an ampholyte with pKa of 2.5 (−COOH group) and 7.3 (−NH_2_ group) [62], and shows the lowest solubility at the isoelectric pH: 4.9 [64]. Upon mixing with TPN_aq_ the pH is well above the pI, which indicates that the observed precipitation should be from calcium phosphate.

Ondansetron hydrochloride has a pKa value of 7.4 and is soluble in water, but the solubility decrease when pH is > 5.7 [62]. Precipitates can form at pH 5.7 and 7, but it has been reported that the precipitate can be re-dissolved at pH 6.2 [56]. The pH of commercially available ondansetron solutions are adjusted with acidic buffer to be in the range of 3.3 to 4 [56]. The measured pH values of Kabiven® and SmofKabiven® mixed with ondansetron were below 5.7 and the pH value of Olimel® N5E was around 6.2. These findings support the fact that no signs of precipitation were observed with ondansetron in the current study.

A situation where the prediction of compatibility is not straightforward can be illustrated with paracetamol. Paracetamol’s phenol group has a pKa of 9.7 [63], which mean it is a neutral molecule below this pH. The formulations’ pH-values are on the acidic side (pH 4.5 up to 5.5-7.0 (Table 3)), which after mixing with TPN_aq_ was found to approach the pH of the respective TPN products. Paracetamol is very slightly soluble in cold water [62]. A typical formulation of paracetamol has a concentration of 10 mg/ml, and the presence of mannitol (Table 3) probably acts as a co-solvent. Therefore, two scenarios might be relevant when mixing with TPN in Y-site; 1) the solubility of paracetamol is aided through dilution into a larger volume or 2) the concomitant dilution of mannitol might decrease the solubility leading to precipitation of paracetamol. It is difficult to predict the outcome on a theoretical level. Other excipients in the paracetamol formulations might also affect Y-site compatibility, and in the current study three generic drugs of paracetamol were investigated. Excipients are stated in the manufacturers SmPC, but not quantitatively (Table 3). Two of the formulations contained cysteine, which might precipitate with copper [9]. This might be a problem when mixing these formulations with TPN containing a lot of trace elements. One of the formulations contained disodium phosphate dihydrate. This might increase the risk of calcium phosphate precipitation, especially if mixed with a TPN product already added maximum electrolytes. To better predict compatibility it would be helpful to know the exact amount of excipients in the formulations. Furthermore, one of the formulations contained hydroxyethyl starch. Some etherified starches (hetastarch) are incompatible with many compounds [65], and might be a factor to take into consideration.

Theoretical considerations of the drugs’ pKa value (s), functional groups, solubility and excipients, together with pH-measurements are important tools in predicting compatibility [64], and should always be an essential part in compatibility assessments. Because theoretical evaluations are not necessarily straightforward, a combination of theory and experiments is advisable.

### Emulsion stability analysis

Methods suitable to assess the emulsion stability upon mixing TPN with drugs should be able to detect changes in the droplet size. Early signs of destabilization can be recognized as an increase in the number of large diameter droplets, and at a later stage a shift of the droplet size distribution towards larger droplets. The results from the emulsion stability analysis can be seen in Table 6.

**Table 6:** Results on emulsion stability

TPN	Drug	Time after mixing *h*	*Laser diffraction*					*Dynamic light scattering*		*Light obscuration*			ζ (*mV*)	*pH* ^d^
			^a^% < *500 nm* ^b^	^a^% < *1* μ*m* ^c^	^a^% > *5* μ*m* ^c^	^a^% > *10* μ*m* ^b^	*V.W. MDD nm*	*I.W. MDD nm*	*PI*	*PFAT2*	*PFAT5*	*PFAT10*		
Olimel® N5E	Fresh without drug	-	91	100	0	0	330 ± 0	273 ± 2	0.05 ± 0.03	0.04 ± 0.01	0.02 ± 0.01	0.01 ± 0.01	−38 ± 3	6.38
	Heated 2 days (45 °C)	-	89	100	0	0	344 ± 2	266 ± 2	0.06 ± 0.01	5.43 ± 0.24	4.47 ± 0.22	1.17 ± 0.08	−38 ± 1	6.27
	Heated 4 days (45 °C)	-	68	75	9	5	1531 ± 25	275 ± 2	0.09 ± 0.03	5.60 ± 2.60	4.55 ± 2.26	2.19 ± 1.26	−41 ± 4	6.17
	Ampicillin	0	91	100	0	0	326 ± 1	272 ± 3	0.05 ± 0.02	0.05 ± 0.02	0.03 ± 0.02	0.02 ± 0.02	−51 ± 1	7.95
		4	92	100	0	0	326 ± 15	270 ± 1	0.05 ± 0.02	0.04 ± 0.02	0.02 ± 0.02	0.02 ± 0.02	−53 ± 2	7.83
	Ondansetron	0	90	100	0	0	325 ± 1	273 ± 2	0.06 ± 0.02	0.05 ± 0.00	0.03 ± 0.00	0.01 ± 0.01	−35 ± 2	6.17
		4	91	100	0	0	322 ± 0	272 ± 3	0.06 ± 0.02	0.04 ± 0.00	0.02 ± 0.01	0.01 ± 0.01	−41 ± 1	6.16
	Paracetamol	0	90	100	0	0	322 ± 0	274 ± 2	0.09 ± 0.01	0.04 ± 0.00	0.02 ± 0.00	0.01 ± 0.00	−43 ± 2	6.21
		4	91	100	0	0	318 ± 0	274 ± 7	0.06 ± 0.01	0.04 ± 0.01	0.02 ± 0.01	0.01 ± 0.00	−38 ± 2	6.20
Kabiven®	Fresh without drug	-	82	100	0	0	365 ± 0	287 ± 11	0.11 ± 0.04	0.22 ± 0.04	0.12 ± 0.03	0.05 ± 0.03	−37 ± 2	5.55
	Heated 2 days (45 °C)	-	76	100	0	0	393 ± 0	287 ± 2	0.10 ± 0.01	6.35 ± 1.25	5.06 ± 1.04	1.65 ± 0.32	−39 ± 2	5.40
	Heated 4 days (45 °C)	-	66	81	9	5	1616 ± 27	316 ± 19	0.25 ± 0.10	6.67 ± 0.22	5.48 ± 0.42	3.01 ± 0.89	−38 ± 3	5.48
	Ampicillin	0	83	100	0	0	353 ± 0	275 ± 2	0.07 ± 0.03	0.12 ± 0.01	0.06 ± 0.01	0.02 ± 0.01	−51 ± 1	7.92
		4	83	100	0	0	359 ± 0	278 ± 1	0.09 ± 0.01	0.10 ± 0.02	0.06 ± 0.02	0.04 ± 0.02	−51 ± 3	7.82
	Ondansetron	0	83	100	0	0	355 ± 0	280 ± 2	0.07 ± 0.01	0.11 ± 0.01	0.06 ± 0.01	0.02 ± 0.00	−37 ± 1	5.55
		4	84	100	0	0	346 ± 0	280 ± 2	0.09 ± 0.02	0.13 ± 0.00	0.06 ± 0.00	0.02 ± 0.00	−37 ± 1	5.55
	Paracetamol	0	82	100	0	0	364 ± 0	277 ± 1	0.09 ± 0.02	0.11 ± 0.00	0.05 ± 0.00	0.02 ± 0.00	−38 ± 2	5.63
		4	83	100	0	0	357 ± 0	281 ± 2	0.07 ± 0.00	0.13 ± 0.00	0.07 ± 0.02	0.03 ± 0.01	−40 ± 2	5.60
SmofKabiven®	Fresh without drug	-	89	100	0	0	326 ± 0	248 ± 1	0.06 ± 0.01	0.31 ± 0.13	0.22 ± 0.11	0.06 ± 0.04	−34 ± 2	5.43
	Heated 2 days (45 °C)	-	66	84	5	2	980 ± 11	279 ± 4	0.15 ± 0.03	5.65 ± 1.07	4.51 ± 1.01	1.79 ± 0.81	−33 ± 3	5.39
	Heated 4 days (45 °C)	-	22	41	30	14	4083 ± 75	342 ± 10	0.29 ± 0.03	8.69 ± 0.97	6.97 ± 0.84	3.21 ± 0.45	−38 ± 3	5.36
	Ampicillin	0	89	100	0	0	324 ± 0	242 ± 2	0.08 ± 0.02	0.35 ± 0.25	0.27 ± 0.22	0.10 ± 0.10	−48 ± 2	7.52
		4	90	100	0	0	317 ± 0	242 ± 1	0.08 ± 0.01	0.22 ± 0.10	0.14 ± 0.08	0.04 ± 0.02	−49 ± 2	7.34
	Ondansetron	0	89	100	0	0	324 ± 0	249 ± 1	0.08 ± 0.01	0.37 ± 0.16	0.27 ± 0.14	0.08 ± 0.05	−37 ± 2	5.54
		4	89	100	0	0	330 ± 1	247 ± 1	0.08 ± 0.02	0.57 ± 0.08	0.39 ± 0.07	0.11 ± 0.05	−34 ± 1	5.56
	Paracetamol	0	89	100	0	0	324 ± 0	242 ± 2	0.08 ± 0.02	0.38 ± 0.27	0.28 ± 0.25	0.12 ± 0.13	−39 ± 2	5.34
		4	90	100	0	0	325 ± 1	244 ± 7	0.11 ± 0.04	0.83 ± 0.58	0.44 ± 0.22	0.12 ± 0.08	−40 ± 4	5.34

a: volume weighted % of oil droplets; b: standard deviation 0–2 c: standard deviation 0–1; d: standard deviation 0.00-0.06

*V.W. MDD* volume weighted mean droplet diameter, *I.W*. intensity weighted, *PI*, polydispersity index, Mean ± standard deviation: *n* = ≥ 3, dynamic light scattering, *n* = 2 with multiple runs, laser diffraction, *n* = 1 with multiple runs

### Dynamic light scattering

All TPN products tested showed I.W. MDD well below 500 nm and comply with the USP requirements; Kabiven® had the largest I.W. MDD with 287 ± 11 nm and SmofKabiven® the smallest with 248 ± 1 nm (Table 6). Based on the DLS results, the MDD of Olimel® N5E did not change during heat destabilization, but for Kabiven® and SmofKabiven® it increased slightly after four days of heating, but were still well below 500 nm. Only minor changes were detected in MDD after mixing with drugs for all the investigated TPN products. However, since the heat destabilized samples were not properly recognized, it strongly suggests that DLS is not suitable for assessing emulsion stability of TPN. The changes in MDD as result of an increase in large diameter droplets were too small to provide reliable data.

PI provides information to the broadness of the droplet size distribution; a PI above 0.50 indicates a very broad size distribution whereas values around 0.10 suggest a relatively monodisperse distribution [66]. A broadening of the droplet size distribution could reflect increased droplet sizes in the large diameter tail of the emulsion, but the PIs measured in the current study were below or around 0.10, only heat destabilized Kabiven® (four days) and SmofKabiven® (two and four days) showed slightly higher PIs. This suggests that also not PI is a suitable measure for the early signs of destabilization of TPN emulsions.

DLS is most suitable for detection of small droplets, in the nanometer size-range, and less accurate for droplets with diameters above 1 μm, which are the most interesting for stability assessment of the TPN products. This makes the recorded MDD and PI less suitable for stability assessment of TPN emulsions. DLS should therefore not be the method of choice for compatibility testing.

### Laser diffraction

LD has a wider measuring range and should therefore be better suited for the detection of the larger diameter droplets. The V.W. MDD of all TPN products showed a significant increase in droplet size after four days of heating (Table 6), and all were above 500 nm. The MDD had also increased after two days, but this was less pronounced for Olimel® N5E and Kabiven®, and was probably in range of the normal variation: e.g. for Olimel® N5E the MDD had increased from 330 to 344 nm, an *increase* of 4 % after two days of heating. No increase in MDD was detected after mixing with the drugs, and all were below 500 nm. Scrutinizing the different size fractions one could see a tendency towards a decrease in the percent of droplets below 500 nm after two days of heating for all TPN products. An increase in number of droplets above 5 μm was observed in the four day samples for all TPN products. The most pronounced changes were observed in SmofKabiven® after four days of heating; both the decrease in droplets below 500 nm and the increase in droplets above 5 and 10 μm. This suggests that the method could offer some support in the detection of destabilization. However, LD does not appear to be a very sensitive method since large diameter droplets were only detected in the most destabilized samples. Also, no droplets above 1 μm were detected in any of the mixed drug: TPN samples. This seems unlikely since some droplets are normally present in the large diameter tail, even in stable emulsions. The limitations of ensemble techniques such as DLS and LD in detecting growth in the population of large oil droplets have been discussed by several researchers [42, 47, 53, 67, 68]. Nevertheless, LD ranked the stable and destabilized emulsions in the expected order, and the method might offer some support in finding at least the most destabilized samples.

### Light obscuration

**Fig. 4:**
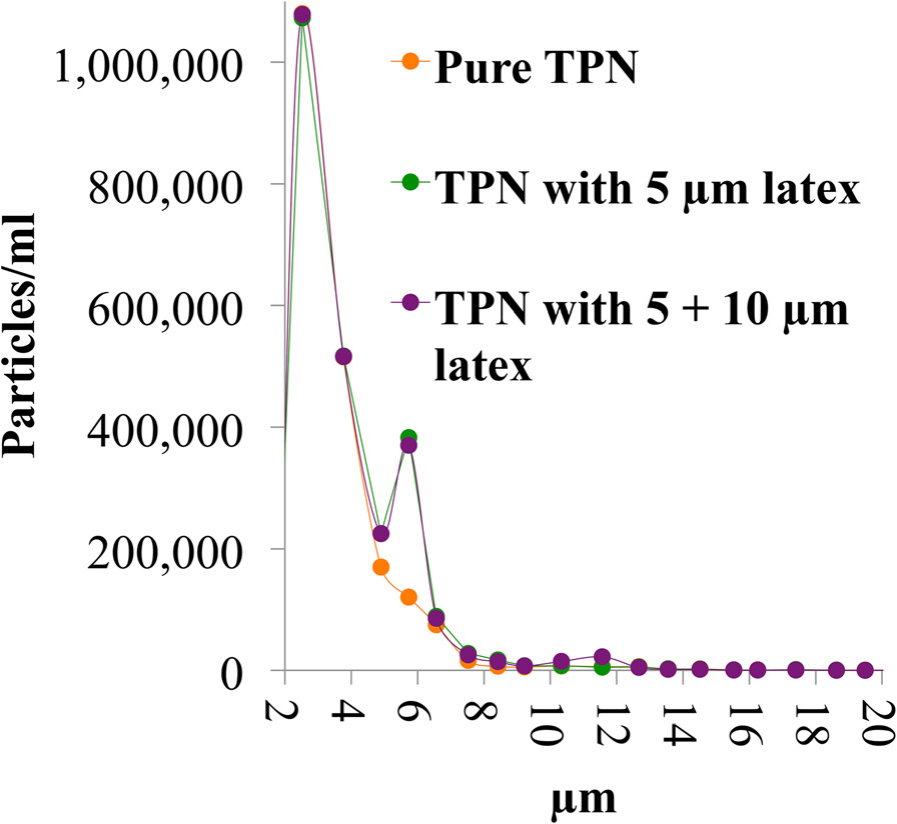
Response of the light obscuration instrument to TPN without (orange) and spiked with polystyrene size standards of 5 μm (green) and 5 + 10 μm (purple) respectively

Significant increases in PFAT values were observed after heat-destabilization of TPN samples, both after two and four days. The difference between two and four days was not significant due to large variation, but the mean values placed the samples in the correct order of stability (larger mean in four day samples), indicating continuous droplet growth under the stressful conditions. LO has previously been proved superior to LD in detecting unstable emulsions and emulsions spiked with latex particles [42, 47]. As a control a similar test as described by Driscoll et al. [47] was performed; TPN samples spiked with 5 and/or 10 μm polystyrene size standards, which turned out not to be detectable by LD (data not shown), was detected by LO (Fig. 4). This confirmed that LO is more sensitive for detecting large diameter droplets and early signs of emulsion destabilization.

A drawback of LO, and several other particle sizing methods (also DLS and LD), is the extensive dilution necessary to avoid coincidence of multiple particles, and this might influence the emulsion characteristics [8]. Furthermore, some of the PFAT values in the current study showed rather large variation, as can be seen in the large standard deviations. This might be attributed to low sampling volume, and an automatic dilution system [46] would allow testing of larger sample volumes, which might contribute to reduce the variation between replicates.

All three TPN products showed PFAT5 values below 0.40 % before addition of drug. SmofKabiven® had the highest PFAT5 followed by Kabiven® and Olimel® N5E. Many factors affect the stability of these multi component systems, like packaging, manufacturing process and raw materials in addition to the composition [8, 44, 45, 69]. PFAT5 of Kabiven® and Olimel® N5E remained low after mixing with drugs, whereas for SmofKabiven® PFAT values were generally higher, and PFAT5 were sometimes found to be above 0.40 %. The PFAT5 values of paracetamol: SmofKabiven® and ondansetron: SmofKabiven® were significantly higher after 4 h compared to pure SmofKabiven®.

A difference in PFAT of heat destabilized TPN was detected depending on container material (data not shown). Higher PFAT-values were found for samples stored in glass than the same samples stores in plastic. This has also been discussed in the literature; large oil droplets might adhere or be absorbed to plastic containers over time [70]. Gonyon and colleagues found an approximately 20 % decrease in PFAT5 for TPN emulsions stored for 6 h (ambient temperature) in plastic, compared to almost 0 % loss when stored in glass [70]. Since plastic sample tubes were used in the current study, a conservative approach might be to add 20 % on the PFAT5 numbers as a precaution. Doing this, the conclusions do not change. Glass might be a more appropriate material for testing the emulsion stability, although choosing plastic might better simulate the infusion environment; plastic is often the material of infusion containers and certainly infusion tubing.

### Microscopy

As expected, the majority of the droplets were very small, and too small to be properly identified using a conventional LM method. Therefore the determination of the MDD was not possible. Only larger droplets (>1 μm) could be counted. Due to the many layers of droplets and the droplet flattening during sample preparation, it was challenging to obtain the proper adjustment of the focus, and to interpret the images. The number of droplets above 2 μm and 5 μm were compared in pure, non-treated and destabilized TPN samples (*n* = 5 images per sample). This provided the appropriate ranking of stable and heat destabilized samples, but the number of large droplets counted varied a lot between the different pictures (data not shown). Also the drug: TPN samples showed large variation in the number of large droplets, and it was difficult to draw any conclusions based on this assessment (data not shown).

Several factors might improve the outcome of microscopy, such as larger sampling volumes, use of proper contrasting technique, use of image analysis programs and more training. LM has been extensively used to investigate stability of parenteral emulsions [13, 28, 36, 37, 40], and was found to be most sensitive in detecting large oil droplets compared to LD, DLS and coulter counter by Müller and Heinemann [36]. It is further emphasized that presence of flocculation can be seen by microscopy [8], and thus allow the distinction between flocs and coalesced droplets. It might also be a more readily available technique in e.g. a hospital pharmacy setting [28, 36, 59]. Nevertheless, in our experience it requires extensive training and experience to ensure the quality and usefulness of microscopy data. It is also time-consuming and laborious, because of the number of samplings necessary and due to the counting process. Driscoll et al. found a modest degree of correlation between LM and LO, and suggests microscopy only as a support to LO [40]. Only a few droplets per field are in focus and counted, therefore analysis and statistical depiction of polydisperse samples is very difficult [49]. Problems of obtaining representative sampling as well as statistics are further discussed in literature [28, 40, 49].

### pH-measurements and theoretical consideration

Olimel® N5E measured the highest pH value of the TPN products followed by Kabiven® and SmofKabiven®. It appeared to be a reasonable correlation between the pH of the TPN products and their respective PFAT5 values; the lower the pH the higher PFAT5. Evaluation of pH and other factors (ionic strength, dilution etc.) might help to understand and predict emulsion instability upon mixing with drugs, however, droplet size measurements should be performed.

Similar to the TPN_aq_ measurements, the pH values did not change much after addition of drugs, with the exception of ampicillin. There were some small differences between pH values measured in drug: TPN and drug: TPN_aq_, mean differences of 0.01-0.15 units (Table 5 and 6). However, these small differences seem less important and should not imply a notable different risk of precipitation in TPN versus TPN_aq_.

### Zeta potential measurements

It is known that the pure lipid injectable emulsion typically has a zeta potential of–30 to–50 mV, due to the negatively charged phospholipids [8]. This charge causes a mutual repulsion between emulsion droplets, keeping them separated and stabilizing the emulsion. At zero potential there is a great risk of emulsion cracking. Because of the presence of many positively charged ions, the zeta potential in TPN is much smaller than in pure lipid emulsion [8, 34, 71]. Different dilution media are reported in the literature for measuring zeta potential of TPN; some dilute in distilled water [25, 28, 38], while others compose an identical TPN aqueous-phase without the lipids and use this as dilution medium [8, 34, 37, 71]. Since diluting in the composed TPN water-phase would give zeta potentials around−/+ 1–2 mV, and these small potentials are in the same order of magnitude as the accuracy of the instrument [8], it may be difficulty to elicit effects on the zeta potential of TPN. Sample heating can also be a problem. Therefore, dilution with water was chosen in the current study, although this means comparing relative differences and not the true values.

The zeta potential of drug: TPN mixtures is a result of several factors. Cations (e.g. Ca^2+^, Na^+^, K^+^ and H^+^) pull the zeta potential closer to zero by non-specific and specific adsorption to the droplet surface [8, 71]. Therefore, drugs diluted in NaCl might be more likely to affect the zeta potential, than drugs diluted in glucose. Glucose, although acidic, is not expected to have a large effect on the zeta potential of TPN because of the buffering effect of the amino acids [8, 34]. All the drugs, except ampicillin, were mixed in 5 % glucose. The zeta potential might also be affected by the pH of the drug solution or the drug molecule itself.

Generally, the zeta potentials of the tested drug: TPN combinations were in the range –30 to –40 mV, only ampicillin was standing out with a zeta potential around –50 mV with all TPN products. The alkaline pH of ampicillin (pH 8.87), can explain the more negative potential. None of the other drugs seemed to greatly affect the zeta potential, even though deviations from the original samples were recorded. The minimal changes in pH after mixing with drug support this finding.

Diluting in water does not provide the real potential and thus no information on how close to zero the potential really is, as pointed out above. It was therefore difficult to assess emulsion stability from this and therefore zeta potential measurements alone are not suitable for compatibility assessments. However, it might support the understanding of what is going on in the mixtures–at least the direction of the charge, but this information could also be inferred from the pH-measurements.

### Discussion of test program and compatibility testing

All the characterization methods used in the current study have their strengths and weaknesses. Investigating compatibility of drugs and complex systems, such as TPN admixtures, requires testing with multiple methods and a thorough evaluation of several indicators of incompatibility to draw safe conclusions. The collective review of result from a panel of qualified methods can provide more robust data; therefore, the statistical tests were limited to investigations into the separate methods and not used for the overall compatibility evaluation.

For the assessment of potential precipitation the total evaluation of sub-visual particle counts (LO), turbidity, visual examinations using Tyndall light and pH measurements made it possible to indicate the presence of precipitates. They all captured different indications of precipitation and should be used together to increase the chance of detecting incompatible blends.

For the analysis of emulsion stability, LO followed by estimation of PFAT5 seemed to be the most sensitive method, since it individually counts the droplets in the large diameter tail [47]. It also gave the most marked response following the droplet distribution from the original samples to heat destabilized TPN in this study. Furthermore, LO is the method recommended by the USP for investigating the large diameter tail of lipid injectable emulsions [41]. A drawback of LO was its low repeatability at times. LD seemed more robust in this sense. We suggest LD, pH measurements and theoretical evaluation as complementing methods to LO for testing emulsion stability. The DLS did not provide additional information on mean droplet size, and was not sensitive enough to detect small changes in the large diameter tail. The LM method used was sub-optimal, but with appropriate adjustments and more experience this might provide useful support. Measuring zeta potential did not add information that could not be extracted from the pH measurements, which is simpler to perform.

The compatibility data provided in the current study are supported by scattered reports in literature, further founding the suitability of the test program. However, conflicting data are common, which complicates comparison to existing literature. Also in the current study conflicting data was obtained, as some of the drugs were compatible with some of the tested TPN admixtures, but not with all. Conflicting results might occur even in a well-controlled experiment. This emphasizes the risk of extrapolation of results from one product to another, and can be equally relevant for generic drug products as for different TPN products. We believe that always using an in-line-filter, provided the drug can be filtered, is a useful safety measure to protect the patient during Y-site administrations.

### Discussion of acceptance criteria in compatibility testing

It is challenging to define definite numerical limits for each of the method for what should be considered a true incompatibility involving these complex blends. The acceptance criteria should relate to safety, and one reason why it is challenging to define absolute criteria is that it is not fully clear how much the body can handle of particles and enlarged oil droplets, both when it comes to sizes and numbers, and therefore it is unclear what the methods should detect at a minimum. Even though a change might be detected it might not be a relevant change with clinical significance. Furthermore, some of the results are difficult to interpret. Take counting of sub-visual particles as an example: Our experience is that for some samples the initial counts can be somewhat increased as compared to the controls but after four hours they are much lower. This could be a sign of a temporary, local precipitate forming (because of concentrated layers), which dissolves with time when the sample gets more homogenous. Or, it can be an effect of aggregation of small particles into fewer large particles or crystal growth (Ostwald ripening) with the same result. It can also be a result of bubbles or other artifacts. This can be hard and time consuming to elucidate.

It is also difficult to find suitable standards for testing and validation of the methods. Using e.g. polystyrene particles provides some information, but since these particles are very different from both precipitates and oil droplets, it might not be transferable to real samples. Therefore, we have used samples that were expected to be closer to real precipitations (forced calcium phosphate precipitations) and destabilized samples (heat stressed) for testing of the methods. It is important to notice that the “incompatibility” in these samples is not always reproducible, so to use them as references to determine the acceptance limit is complicated. But then again, the nature of the incompatibility reaction may also vary from time to time in a real setting. The timing and extent of precipitation can differ even for identical samples investigated in the same study [31]. Several factors that are not easily controlled can influence the question of compatibility; e.g. the temperature, light conditions, different batches of tested products etc.

Even though acceptance limits are difficult to define, some measure of what is a large or a small change compared to controls is important in order to interpret the results. Based on the current experiences we suggest some arbitrary limits for each method of the test program: 1) Visual examination: No clear signs of particles or increased Tyndall effect should be detected. 2) LO: It is difficult to set a fixed limit for what is an alarming increase in sub-visual particles. Particle counts above 0.5 μm approaching 1000–2000 particles/ml (provided that both drug and TPN are filtered 0.22 μm before mixing) is approximately 10–20 times the background count of TPN_aq_, and should be a reason to react. It is also important to look for growth of larger particles (c.f. Ph. Eur. limits) and to see how the particle counts develops over time. If there is no clear increase over time, either in number or particle size, it might be a compatible blend. Noteworthy, in ampicillin: TPN_aq_ (Table 5) samples, and TPN_aq_: NaOH, the Ph. Eur. limits were not exceeded, even though a large increase in smaller particles (e.g. from 485/ml immediately to 9550 particles/ml ≥ 0.5 μm after four hours for ampicillin: Olimel® N5E) indicated precipitation. These small particles might grow and aggregate with time and constitute a risk if infused. This suggests that using the Ph. Eur limits only, may not be sufficient when it comes to dynamic situations as precipitation and compatibility studies. For the evaluation of “static” particles such as dust, fibers, glass etc. the Ph. Eur. limits are more applicable. 3) Turbidity: Attention should be payed to turbidity values > 0.20-0.30 FNU, although this has to be seen in comparison to the background turbidity of both TPN_aq_ and drug. Based on experience with incompatible blends that did not exceed the currently applied limit of 0.5 FNU, a lower level might be suitable, and results from complementing methods will be required for reliable judgment of compatibility. However, we have occasionally measured Milli-Q-water as high as 0.18 FNU. Care should be taken to avoid disturbing factors like scratches on sample cells, bubbles etc. 4) pH: The pH values should be interpreted in relation to the pKa value of the drug and the pH of the TPN. If mixing moves the pH in direction of less ionized drug, the solubility decreases. It is also alarming if the pH approaches 7 with regard to calcium phosphate precipitation, and if it is below 5.5. The latter is due to phospholipids being ionized at pH values 5.5-9.0 [33]. At pH values below this, the charge might start to neutralize, increasing the risk of emulsion destabilization. However, some phospholipids are not completely neutralized before reaching pH 3.2 [8], therefore the emulsion might stand lower pH values than 5.5. 5) PFAT5: It is difficult to define an alarming increase based on the current data, and we therefore lean on the PFAT5 limit of < 0.40 %. 6) LD: MDD should be below 500 nm as is the requirement in the USP. Like PFAT5, it is difficult to define an alarming increase. Bouchoud et al. suggested a limit of less than 10 % change in MDD measured by DLS as evidence of compatibility [13], which helps assuring that the MDD is below 500 nm. In addition the size fractions should be investigated, and there should be no increase in sizes above 5 μm. Finally, to answer the ultimate question whether “to mix or not to mix”, the responses from all method have to be seen together, and combined with theoretical considerations. More work and discussions on methods and suitable acceptance criteria for compatibility testing is welcomed.

## Conclusion

A test program suitable for investigating *physical* compatibility of drugs and TPN mixed at Y-site was established and evaluated. The milk-white appearance of TPN admixtures prohibits the assessment of potential precipitation, and a sample preparation method replacing the lipid phase with Milli-Q-water was recommended for these experiments. Light obscuration, turbidimetry, visual examination using Tyndall beams, and pH-measurements were selected methods for detecting signs of precipitation. To investigate signs of emulsion destabilization, samples containing the complete TPN admixture were used. Among the tested methods light obscuration followed by estimation of PFAT5, laser diffraction and pH-measurements were found to be the most adequate. It should be emphasized that none of the methods should be used alone. The different methods supply information of complementary descriptors of compatibility and should be seen together. Mixed samples should always be compared to pure controls and the samples should be followed over a period of time. Theoretical prediction is important to understand and support the experimental tests. Acceptance criteria are difficult to define, although some critical limits are suggested based on current experience.

## Abbreviations

DLS: dynamic light scattering
FNU: formazin nephelometry units
I.W.: intensity weighted
LD: laser diffraction
LM: light microscopy
LO: light obscuration
MDD: mean droplet diameter
NTU: nephelometric turbidity units
PFATX: volume weighted percentage of fat greater than X microns
PI: polydispersity index
TPN: total parenteral nutrition
TPN_aq_: TPN where lipid emulsion is replaced by Milli-Q-water
W.V.: volume weighted
